# Identification of New Players in Cell Division, DNA Damage Response, and Morphogenesis Through Construction of *Schizosaccharomyces pombe* Deletion Strains

**DOI:** 10.1534/g3.114.015701

**Published:** 2014-12-31

**Authors:** Jun-Song Chen, Janel R. Beckley, Nathan A. McDonald, Liping Ren, MariaSanta Mangione, Sylvia J. Jang, Zachary C. Elmore, Nicole Rachfall, Anna Feoktistova, Christine M. Jones, Alaina H. Willet, Rodrigo Guillen, Danny A. Bitton, Jürg Bähler, Michael A. Jensen, Nick Rhind, Kathleen L. Gould

**Affiliations:** * Department of Cell and Developmental Biology, Vanderbilt University School of Medicine, Nashville, Tennessee 37232; † Department of Genetics, Evolution and Environment and UCL Cancer Institute, University College London, London WC1E 6BT, United Kingdom; ‡ Genome Technology Center, Stanford University School of Medicine, Palo Alto, California 01605; § Department of Biochemistry and Molecular Pharmacology, University of Massachusetts Medical School, Worcester, Massachusetts 01605

**Keywords:** fission yeast, DNA metabolism, cell division, gene deletion, stress sensitivity

## Abstract

Many fundamental biological processes are studied using the fission yeast, *Schizosaccharomyces pombe*. Here we report the construction of a set of 281 haploid gene deletion strains covering many previously uncharacterized genes. This collection of strains was tested for growth under a variety of different stress conditions. We identified new genes involved in DNA metabolism, completion of the cell cycle, and morphogenesis. This subset of nonessential gene deletions will add to the toolkits available for the study of biological processes in *S. pombe*.

Large-scale genetic screening is a particularly effective means to establish a wiring diagram of functional relationships for genes of unknown or redundant function (Costanzo *et al.* 2011). This approach was pioneered in the budding yeast, *Saccharomyces cerevisiae* (Dixon *et al.* 2009), and has also been used successfully in the fission yeast, *Schizosaccharomyces pombe*, to identify genes involved in cell cycle control (Navarro and Nurse 2012; Hayles *et al.* 2013), cell size (Kelly and Nurse 2011), sporulation (Ucisik-Akkaya *et al.* 2014), TORC1 inhibition (Rallis *et al.* 2014), valproic acid sensitivity (Zhang *et al.* 2013), cobalt homeostasis (Ryuko *et al.* 2012), resistance to TORC1 inhibition (Rallis *et al.* 2014), cadmium tolerance (Kennedy *et al.* 2008), micafungin sensitivity (Zhou *et al.* 2013), the DNA damage response (Pan *et al.* 2012), chromosome cohesion (Chen *et al.* 2012), and other processes.

The majority of the aforementioned *S. pombe* genetic screens used a haploid strain collection containing 3000–3300 protein coding gene deletions constructed by and obtained from Bioneer Corporation (Republic of Korea). This valuable collection represents a significant portion of the known 3576 nonessential protein-coding genes (Kim *et al.* 2010). At the time this ambitious deletion project began, it was estimated that 4914 protein-coding genes existed in the *S. pombe* genome. Since then, additional protein-coding genes have been identified or predicted (Hayles *et al.* 2013; Bitton *et al.* 2011), and there are currently 4981 protein-coding genes annotated for *S. pombe* (http://www.pombase.org/) (Wood *et al.* 2012). It follows, then, that large-scale genetic screens performed thus far did not include all possible viable *S. pombe* gene deletions.

To fill this gap in available gene deletion strains, we constructed 281 haploid gene deletion mutants that were not a part of the approximately 3000 gene deletion strains available as version 3 from Bioneer Corporation. We conducted growth assays for all these deletions under different environmental stresses and report new players in the processes of DNA metabolism, cell division, and morphogenesis.

## Materials and Methods

### Yeast strains, media, and materials

The parent strain used for constructing the gene deletions was *ade6-M210 ura4-D18 leu1-32 h+* (KGY247). Deletions containing *rad22-GFP*::*kan^R^* were made by crossing the deletion strains with a lab stock strain *rad22-GFP*::*kan^R^ ade6-M210 ura4-D18 leu1-32 h*- (KLG16715) and performing tetrad analysis. All chemicals were from Sigma-Aldrich (St. Louis, MO) unless otherwise noted.

### Gene deletions

**Figure 1:**
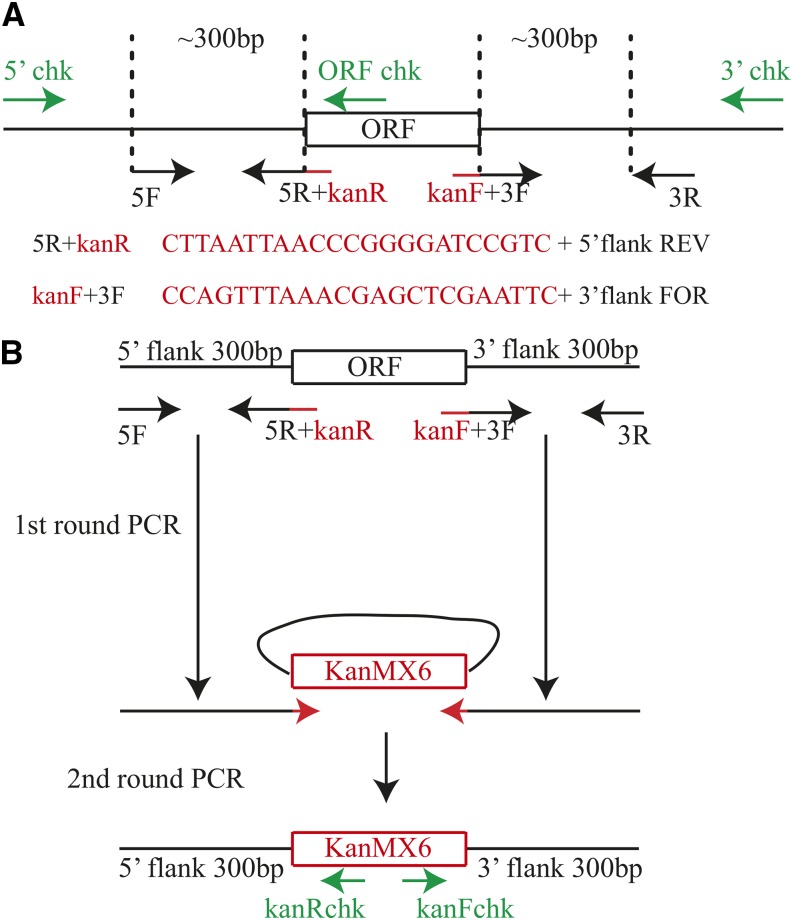
Schematic of gene deletion strategy. (A) The diagram shows the position of the gene specific oligonucleotides used in PCR reactions to make and check the deletions. 5F/5R and 3F/3R were used for amplifying the flanking sequences of the ORF to be deleted. 5′ chk, 3′ chk, and ORF chk were used to validate the successful insertion of the *kanMX6* cassette and deletion of the ORF. (B) Schematic of the process of making the DNA fragment used for deletion of the ORF by homologous recombination. The first round of PCRs produced the 5′ and 3′ flanking sequences of the ORF. The second PCR reactions were performed using the first round PCR products as primers and the *kanMX6* cassette as template to amplify the cassette with gene-specific flanks. The resulting PCR product was used for cell transformation and homologous recombination to complete gene deletion. The positions of the common checking oligonucleotides within the *kanMX6* cassette are shown.

Gene deletions were accomplished as previously published with some modifications as described below (Wach *et al.* 1994). Primers used to amplify the flanking sequences were designed as shown in Figure 1 and listed in Supporting Information, Table S1 and Table S2. Initially the 5F, 5R, 3F, and 3R 24-bp sequences were extracted from the following positions: 5F, −324 to −300 upstream of the ORF ATG; 5R, −24 to −1 upstream of the ORF ATG (starting with the kan start 23 bp in addition); 3F, +1 of the STOP codon to +24 (starting the Kan end 23 bp in addition); and 3R, +324 to + 300 from the ORF STOP codon. Checking primers 5′chk and 3′chk were designed within 500 bp on either side of the ORF, and ORF chk oligo within 100 bp of the ORF. Next, the GC percentages of all primers were examined. When they were found to be lower than 30%, the 5F and 3R sequences and the three checking sequences were optimized with the software Primer3 (Primer3web version 4.0.0; http://primer3.ut.ee/) (Untergasser *et al.* 2012; Koressaar and Remm 2007) by re-selecting a sequence (between 24 and 35 bp) further upstream or downstream of the original site with at least 30% GC content. In these cases, the length of the flanking sequences changed from the targeted 300 bp to range from 200 to 500 bp and the checking oligonucleotide was also re-designed accordingly to make sure it was outside of the deletion construct. Because the position of the 5R and 3F sequences cannot be changed, oligonucleotide sequences with low GC % were extended up to a length of 35 bp to increase the melting temperature.

Oligonucleotides in 96-well plates were re-suspended in 1 mM Tris, pH 7.4, 0.1 mM EDTA at a final concentration of 100 µM. They were then diluted to 4 µM with nuclease free water in a new 96 well plate; 5 µl each of F and R oligonucleotides were mixed with 25 µl GoTaq Green Master Mix (Promega, Fitchburg, WI) and 225 ng *S. pombe* genomic DNA (KGY246; *ade6-M210 ura4-D18 leu1-32 h^−^)* and nuclease free water to a final volume of 50 µl. The PCR was performed using a T3000 Thermocycler (Biometra) with the following program: 95°, 2 min; 12 cycles of (95° for 1 min, 60° for 1 min, and 72° for 1 min); and 25 cycles of (95° for 1 min, 50° for 1 min, and 72° for 1 min); 72° for 10 min. Then, 5 µl of the PCR product was examined on 0.8% agarose gel. If the product was weak or undetectable, then the PCR was re-run with the annealing temperatures reduced to 53° and 48° in the above two-step program.

### PCR of the *kanMX6* cassette

The second-stage PCR reactions included the following in a total volume of 50 µl: 1 µl each of the first-stage 5′ and 3′ flanking sequence PCR products, 100 ng *pFA6-kanMX6* (Bahler *et al.* 1998), 5 µl 10× Easy A buffer, 4 µl dNTPs (stock is 2.5 mM each), and 0.5 µl (2.5 U) Easy A Hi-Fi polymerase (Agilent Technologies, Santa Clara, CA). The following PCR program was used: 94°, 1 min; 30 cycles of (94° for 1 min, 45° for 5 min, and 72° for 3 min); 72° for 10 min. Then, 5 µl of PCR product was checked on 0.8% agarose gel prior to transformation.

### Yeast transformations

*S. pombe* strain *leu1-32 ura4-D18 ade6-M210 h^+^* was grown in YE media at 32° overnight to mid-log phase. Ten OD of the culture was used for each transformation. Cells were pelleted by centrifugation and washed with 10 ml of TE/LiAc (10 mM Tris, pH 7.6, 1 mM EDTA, 100 mM lithium acetate) twice and then re-suspended in TE/LiAc. For each transformation, 100 µl cells, corresponding to the original 10 OD of culture, were mixed and incubated with 20 µl transforming DNA (the second PCR product) and 40 µg boiled salmon sperm DNA. After incubation at room temperature for 10 min, 260 µl of TE/LiAc/PEG (40% PEG in TE/LiAc) was added, and the cells were vortexed gently and incubated at 32° for 30–45 min; 43 µl of DMSO was then added to each tube, followed by incubation at 42° for 6 min. Cells were centrifuged and washed with 0.5 ml sterile H_2_O before being re-suspended in 5 ml of YE media and incubated at 29° overnight. Then, 500 µl of cells were plated on YE-G418 plates and incubated at 29° for 3–5 d. Each plate was replica plated three times to fresh YE-G418 plates before colonies were checked by PCR.

### Whole cell PCR checking of yeast transformants

Whole cell PCR was used to check and select a colony with correct deletion. For each PCR reaction, 5 µl of 4 µM of both forward and reverse oligos were mixed with 10 µl GoTaq green. The following PCR program was used: 95°, 1 min; 12 cycles of (95° for 45 sec, 60° for 1 min, and 72° for 1 min); 25 cycles of (95° for 45 sec, 52° for 45 sec, and 72° for 1 min). Then, 10 µl of each PCR product was checked by running it on an 0.8% agarose gel.

### Sensitivity/resistance assay

Wild-type and deletion strains were grown in YE in 96-well plates to saturation. Ten-fold serial dilutions of each strain were made and spotted onto minimal medium agar plates (supplemented with adenine, uracil, and leucine) or YE agar plates in the absence or presence of the following additions: brefeldin A, 25 µM; bleomycin, 1 µg/ml; calcofluor white, 0.5 mg/ml; cycloheximide, 10 µg/ml; EGTA, 5 mM; hydroxyurea, 7.5 mM; KCl, 1 M; latrunculin A (Cayman Chemical, Ann Arbor, Michigan), 0.25 µM; methyl benzimidazol-2-yl-carbamate (MBC), 10 µg/ml; methyl methanesulfonate (MMS), 0.01%; sodium dodecyl sulfate (SDS), 0.005%; sorbitol, 1.2 M; and thiabendazole (TBZ), 12.5 µg/ml. Plates were incubated at 29° or other temperatures as indicated for 2–8 d and colonies were imaged with a scanner. To assay strain sensitivity, growth of each strain on rich YE media at 29° was compared with other conditions. If deletion cells grew significantly less when exposed to certain reagents or conditions compared with that strain’s growth on YE alone, the gene deletion strain was scored as sensitive to the reagent. Growth sensitivities were confirmed and classified as “very sensitive” if they did not grow relative to wild-type cells, “sensitive” if deletion cells were growing at approximately two or more dilution factors lower than that of wild-type cells, “resistant” if deletion cells grew better than wild-type cells, or “not sensitive” if deletion cells grew similarly or at one dilution factor of wild-type cells. An example is shown in Figure S1.

### Cluster analysis

Cluster analysis was performed with the open source clustering software Cluster3.0 and visualized using Java Treeview. Both software packages were downloaded from http://bonsai.hgc.jp/~mdehoon/software/cluster/software.htm; 2, 1, 0, and −1 were used to represent “very sensitive,” “sensitive,” “not sensitive,” and “resistant,” respectively. Hierarchical clustering was performed with the complete linkage method.

### Microscopy methods

For protein localization studies, live cells were imaged. For visualization of DNA, cells were fixed with ice-cold 70% ethanol. After washing with PBS, the fixed cells were incubated with PBS containing 5 mg/ml methyl blue (MB) or 5 µg/ml calcofluor white for 15 min on ice. MB-stained cells were then pelleted by centrifugation and re-suspended in PBS before imaging. Calcofluor white-stained cells were washed four times with PBS before re-suspension in PBS prior to imaging. Cells were mixed with DAPI before imaging. One of the two following microscopes were used to collect images: a spinning-disk confocal microscope (Ultraview LCI; PerkinElmer) with a 100× NA 1.40 Plan-Apochromat oil immersion objective and a 488-nm argon ion laser (GFP), or a 594-nm helium neon laser (mCherry and RFP) or HBO 100 mercury lamp (DAPI, methyl blue, and calcofluor), or a personal microscope system (DeltaVision; Applied Precision) including a microscope (IX71; Olympus), 60× NA 1.42 PlanApo and 100× NA 1.40 UPlanSApo objectives, fixed-cell and live-cell filter wheels, a camera (CoolSnap HQ2; Photometrics), and softWoRx imaging software. Images on the confocal were captured on a charge-coupled device camera (Orca-ER; Hamamatsu Photonics) and processed using MetaMorph software (version 7.1; Molecular Devices). Images were analyzed and quantified in Image J and imported into Adobe Illustrator for construction of figures. Bar graphs were created in Microsoft Excel and *p* values were calculated using two-tailed unequal variance Student’s *t*-tests.

## Results

### Gene deletion strategy

To make *S. pombe* gene deletions, two sequential PCRs were optimized to produce a single DNA fragment that was then used to replace each gene with the *kanMX6* cassette (Wach *et al.* 1994). This deletion strategy is schematized in Figure 1. First, primers were designed to amplify from *S. pombe* genomic DNA approximately 300 bp of 5′ and 3′ sequences flanking each open reading frame (ORF) targeted for deletion (see *Materials and Methods* for primer design and Table S1, for primer sequences). Then, 23 nucleotides complementary to the *kanMX6* cassette were added to the reverse primer for the 5′ flank reaction and to the forward primer for the 3′ flank reaction. Usually, amplification of the flanking sequences was robust and a sufficient quantity of the PCR product was obtained (see, for example, Figure S2). A second PCR was then performed to amplify the *kanMX6* module within pFA6-*kanMX6* (Bahler *et al.* 1998) sandwiched by the 5′ and 3′ flanking sequences by using the PCR products from the first round of PCR as primers. Each final PCR product (∼2 kb in length) was transformed into the haploid *S. pombe* strain *ade6-M210 leu1-32 ura4-D18 h^+^* because we were interested in obtaining deletions of only nonessential genes for genome-wide screens; integration of the marker was selected by growth on YE-G418 plates at 29°.

To test whether ORFs had been correctly replaced with *kanMX6*, three PCR reactions were performed on G418-resistant colonies (1–2 per targeted gene). The “left” check PCR reaction was performed with a forward primer corresponding to sequences upstream of the 5′ flanking sequence (5′ chk; Figure 1A) and a reverse primer corresponding to sequences 20 bp downstream of the start codon of the *kan^R^* ORF (kanR chk, Figure 1B; Table S1 and Table S2). The “right” check reaction was performed with a forward primer corresponding to sequences 113 bp upstream of the 3′ end of the *kanMX6* cassette (kanF chk, Figure 1B), and a reverse primer corresponding to sequences downstream of the reverse primer used for 3′ flanking sequence amplification (3′ chk; Figure 1A; Table S1 and Table S2). To make sure the ORF had been removed, a third PCR (ORF check) was performed. This reaction used the same forward primer as that used for “left check” (*e.g.*, 5′ chk) and a reverse primer corresponding to sequences inside the ORF (ORF chk; Figure 1A; Table S1 and Table S2). The absence of a PCR product from this reaction strongly suggested that the ORF had been deleted; to rule out the possibility that a technical issue prevented amplification of a product in the ORF check reaction, a fourth PCR reaction was performed using wild-type *S. pombe* cells and the same set of primers. Using this approach, 281 deletions were successfully constructed (Table S1) from ∼400 attempted. Some of the genes that were unsuccessfully deleted from the haploid strain may be essential. This deletion set of 281 strains is available through the Yeast Genetic Resource Center (http://yeast.lab.nig.ac.jp/nig/index_en.html).

### Stress sensitivities of the deletion strains

Although a large percentage of the deleted genes have characterized or predicted functions, several have no functional assignment (Wood *et al.* 2012). To help determine which biological processes the uncharacterized genes participate in, and to explore additional functions for characterized genes, the growth of each of the 281 deletion strains was assayed in 16 different stress conditions. Specifically, the strains were tested for growth at 29° on rich YE agar containing one of the following 13 compounds: the fungal microtubule destabilizing drugs thiabendazole (TBZ) or methyl-2-benzimidazole-carbamate (MBC) (Yamamoto 1980), the actin cytoskeleton destabilizing drug latrunculin A (Lat A) (Ayscough *et al.* 1997), the DNA replication inhibitor hydroxyurea (HU) (Yarbro 1992; Slater 1973), the DNA damage-inducing drugs bleomycin (Povirk 1996) and methyl methanesulfonate (MMS) (Beranek 1990), the secretory pathway inhibitor brefeldin A (BFA) (Turi *et al.* 1994), the protein synthesis inhibitor cycloheximide, the cell wall or membrane structure compromising agents calcofluor white or sodium dodecyl sulfate (SDS), high salt (KCl), the osmolyte sorbitol, or the calcium chelator EGTA. In addition, cells were tested for growth on rich medium at low (19°) or high (36°) temperatures and at 29° on minimal medium (EMM2) agar plates supplemented with adenine, uracil, and leucine. The sensitivities of the 77 deletion strains whose growth was affected by at least one stress are summarized in Table S3 and the various levels of sensitivity are illustrated in Figure S1.

**Figure 2:**
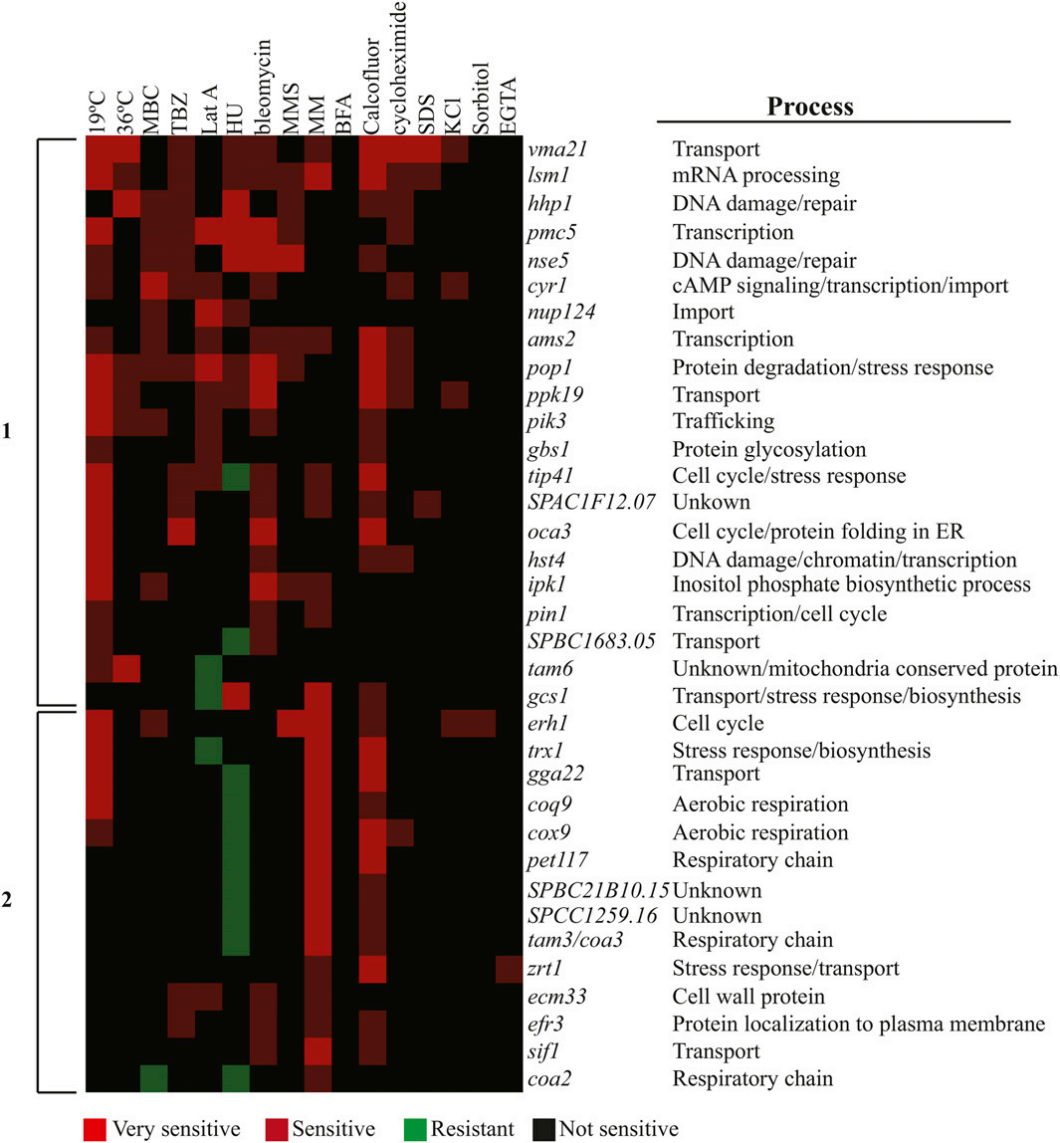
Phenotypic clustering of the deletions that showed sensitivity or resistance to at least three stress conditions. Hierarchical clustering of the sensitivity and resistance patterns of the deletions was performed. GO biological processes of the ORFs involved are shown on the right. Growth conditions are as defined in *Materials and Methods*.

Approximately 12% (35) of the deletion strains showed sensitivity to three or more stresses (Table S4). A survey of the GO functional annotations for the corresponding genes (Wood *et al.*, 2012) linked most to intracellular transport, biosynthesis, cellular metabolism, stress response, and/or transcription (Figure 2). Group 1 genes are involved in intracellular transport, transcriptional regulation, and DNA damage response/repair. Loss of group 1 genes results in pleiotropic effects (*i.e.*, multiple sensitivities), likely because these genes are involved in core cellular functions. Deletions of group 2 genes were characterized by high sensitivity to low nutrient condition (minimal media), calcofluor white, and resistance to HU treatment. Interestingly, several members of this group are involved in biosynthesis and mitochondrial respiration, indicating a strong linkage between respiration and nutrient availability.

### New effectors of cell division

**Figure 3:**
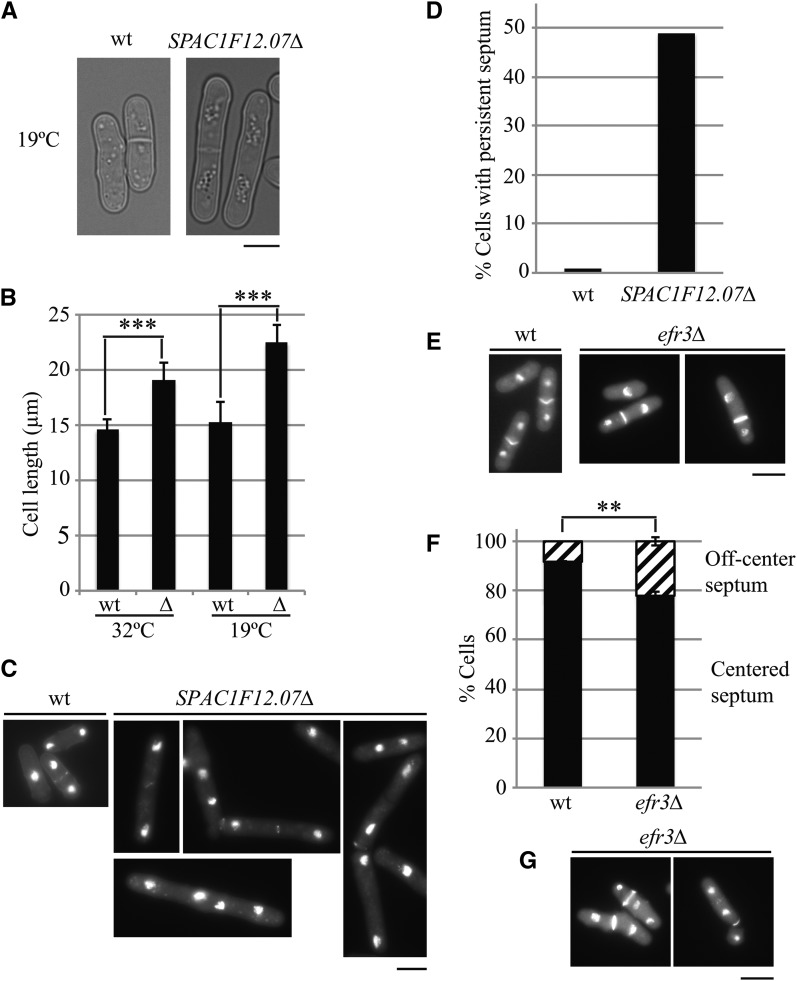
*SPAC1F12.07Δ* and *efr3*Δ cells exhibit cell division defects. (A) DIC images of wild-type (wt) and *SPAC1F12.07Δ* cells. (B) Cell length of wt and *SPAC1F12.07Δ* cells at 32° and 19°. ∆, *SPAC1F12.07Δ*. Standard error of the means (SEM) are shown. ***p < 0.001. (C) DAPI and methyl blue (MB) staining of wt and *SPAC1F12.07Δ* cells. (D) Quantitation of persistent septal material as shown in (C). (E) DAPI and MB staining of wt and *efr3*Δ cells. (F) Quantitation of off-center septum in wt and *efr3*Δ cells. SEM is shown. **p < 0.01. (G) DAPI and MB staining of *efr3*Δ cells showing “cut” phenotype. Scale bars, 5 µm.

Among the 35 strains that exhibited sensitivity to three or more stresses (Figure 2), deletion of two uncharacterized ORFs showed cell division defects that have not been previously reported. *SPAC1F12.07∆* cells were slow-growing at low temperature and sensitive to TBZ, calcofluor white, and SDS, congruent with defects in cell morphogenesis. Microscopic imaging of *SPAC1F12.07∆* cells showed that they were elongated at both 32° and 19° (Figure 3, A and B), and approximately 50% of the cells formed a persistent septum that failed to cleave the daughter cells (Figure 3, C and D); a low percentage of *SPAC1F12.07∆* cells formed no septa at all. *SPAC1F12.07* is predicted to be a phosphoserine aminotransferase involved in L-serine biosynthesis (Wood *et al.* 2012) and peaks in abundance during mitosis (Carpy *et al.* 2014).

Deletion of *efr3* also led to multiple growth sensitivities and defects in cell division (Figure 3, E–G). Staining of the nuclei and cell wall showed that 23% of septating *efr3Δ* cells had a septum misplaced toward one cell tip compared with only 8% of septating wild-type cells, as observed previously (Jourdain *et al.* 2013) (Figure 3, E and F). As a result, some *efr3Δ* cells mis-segregated their chromosomes, displaying a “cut” phenotype, or had variable cell sizes because of asymmetric divisions (Figure 3G). In *S. cerevisiae* and humans, Efr3 is a component of the plasma membrane phosphoinositide (PI) 4-kinase complex that produces PI4P (Wu *et al.* 2014; Baird *et al.* 2008). By analogy, deletion of *efr3* in *S. pombe* likely disrupts PI4P production at the plasma membrane and, therefore, the localization of proteins involved in contractile ring placement and ultimately septum targeting and deposition.

### New players in DNA metabolism

The 10 deletion mutants sensitive to one or more drugs that interfere with DNA metabolism are listed in Table S5. Because the three drugs used differ in their modes of interfering with DNA metabolism, sensitivities also varied. Four gene deletion strains not previously linked with DNA metabolism were analyzed further.

**Figure 4:**
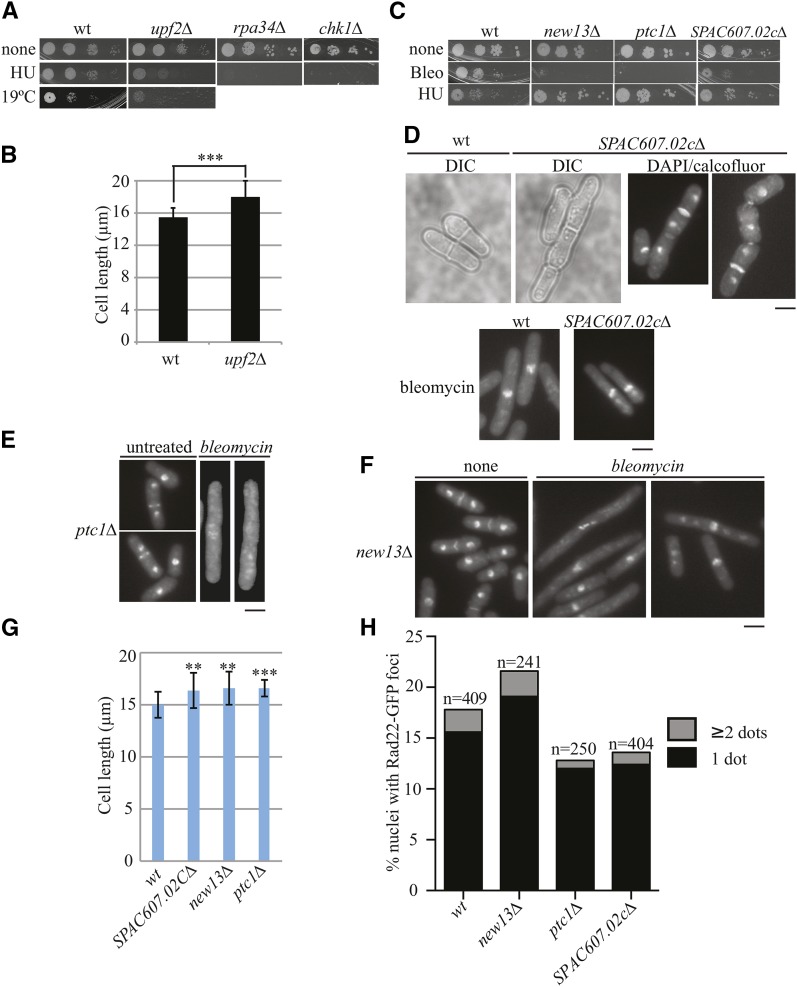
Characterization of gene deletion strains sensitive to DNA damaging agents. (A) The 10-fold dilutions of wild-type, *upf2Δ*, *rpa34∆*, and *chk1∆* cells were spotted on YE agar in the absence or presence of 7.5 mM hydroxyurea (HU). Plates were incubated at 29° and 19° for 3–8 d. (B) The indicated strains were grown at 29° or 19° in liquid YE medium to log phase and the lengths of septating cells were measured with microscopy. SEM is shown. ***p < 0.001. (C) Spotting growth assay of the indicated strains on YE agar in the absence or presence of 1 µg/ml bleomycin (Bleo) or 7.5 mM HU. (D) Images of DIC and DAPI/calcofluor white staining of wild-type and *SPAC607.02cΔ* cells treated (lower panels) or untreated (upper panels) with 1.25 µg/ml bleomycin for 4 hr. (E) DAPI/calcofluor white staining images of *ptc1Δ* cells treated or untreated with 1.25 µg/ml bleomycin for 4 hr. (F) DAPI/calcofluor white staining images of *new13Δ* cells treated or untreated with 1.25 µg/ml bleomycin for 4 hr. (G) Cell length of septated cells of the indicated strains grown to log phase at 32° measured with microscopy. SEMs are shown. **p < 0.01; ***p < 0.001. (H) Quantitation of Rad22-GFP foci in the indicated strains. Scale bars, 5 µm.

As shown previously (Walworth *et al.* 1993; Bimbo *et al.* 2005), deletion of the checkpoint kinase gene, *chk1*, resulted in high HU sensitivity, which served as a control for our experiments (Figure 4A). Two other deletions in our set, *upf2Δ* and *rpa34∆*, were also very sensitive to HU, suggesting an involvement in DNA metabolism (Figure 4A). Rpa34 encodes a subunit of the RNA polymerase I and *S. cerevisiae* Rpa34 interacts with DNA topoisomerase I (Top1) (Beckouet *et al.* 2008). *S. cerevisiae rpa34∆* cells are uniquely sensitive to the overexpression of *TOP1* and are also sensitive to the DNA damaging agent camptothecin (Reid *et al.* 2011). Thus, Rpa34 regulation of rDNA transcription may play a conserved role in DNA repair. *S. pombe* Upf2 has not been well-characterized, but it and its human ortholog (UPF2) form complexes with Upf1/UPF1 and Upf3/UPF3 and participate in nonsense-mediated mRNA decay (Chang *et al.* 2007; Matia-Gonzalez *et al.* 2013). Interestingly, human UPF1, an RNA-dependent and DNA-dependent 5′–3′ helicase, is also involved in DNA synthesis during replication and repair and is required for genome stability (Azzalin and Lingner 2006). The *S. pombe* Upf complex is additionally required for the proper response to oxidative stress (Matia-Gonzalez *et al.* 2013). *upf2Δ* cells are elongated relative to wild-type (Figure 4B), possibly because they contain damaged DNA, which triggers a modest G2/M checkpoint delay.

In contrast to *upf2∆* and *rpa34∆*, deletions of *ptc1* and two uncharacterized genes, *SPAC607.02c* and *new13*, were not overtly sensitive to HU but were very sensitive to bleomycin (Figure 4C). The gene product encoded by *SPAC607.02c* localizes to the nucleolus (Matsuyama *et al.* 2006) and is conserved in fungi but has no apparent *S. cerevisiae* ortholog (Wood *et al.* 2012). A small percentage (2%) of *SPAC607.02cΔ* cells displayed cell separation defects and contained multiple septa (Figure 4D). On bleomycin treatment, *SPAC607.02cΔ* cells arrested in the cell cycle, similar to wild-type cells, indicating an intact checkpoint response. However, unlike in the wild-type, the nuclei of *SPAC607.02cΔ* cells did not remain centered (74% off-center *vs.* 10% in wild-type), and the cells did not become as elongated (Figure 4D).

In deletions of *ptc1*, which encodes a protein phosphatase 2C, 24% of cells exhibited septation and/or chromosome segregation defects exemplified by off-center septa and unequal distribution of the daughter nuclei (Figure 4E). On bleomycin treatment, *ptc1Δ* cells elongated like wild-type cells, indicating an intact DNA damage checkpoint, but the DNA became indistinct or lost in 10% of cells relative to 0% in wild-type (Figure 4E). Although a role for Ptc1 in DNA damage response has not previously been reported, the stress kinase Sty1(Spc1) acts upstream of Ptc1 (Gaits *et al.* 1997), and Sty1 is important for the DNA damage response to oxidative stress (Alao and Sunnerhagen 2008). Thus, Ptc1 might act in this same pathway.

The *new13* ORF overlaps with that of *dsc4*, which encodes a component of a Golgi E3 ligase complex, and *new13* and *dsc4* transcripts converge (Wood *et al.* 2012). Therefore, deleting the entire *new13* ORF removed sequences encoding the C-terminus of Dsc4 and vice versa. In this regard, it is interesting that *dsc4∆* cells are sensitive to the DNA damaging agent, 4-nitroquinoline N-oxide (Deshpande *et al.* 2009), which is similar to the effects of bleomycin. This sensitivity is perhaps due to the concomitant deletion of *new13* rather than loss of Dsc4-dependent Golgi E3 ligase activity. It will be necessary to make smaller deletions in the *new13* and *dsc4* ORFs to dissect their unique roles. *new13∆* cells treated with bleomycin arrested growth like wild-type; therefore, their sensitivity to this agent is not due to a bypass of the DNA damage checkpoint. However, we note that DNA staining became diffuse in 13% of cells, indicating defects in chromatin organization (Figure 4F).

All three of these bleomycin-sensitive deletion strains were elongated relative to wild-type (Figure 4G), a phenotype that could reflect a mild DNA damage-induced checkpoint delay of entry into mitosis. Therefore, we tested whether higher levels of DNA damage exist in *ptc1∆*, *new13∆*, or *SPAC607.02cΔ* cells relative to wild-type using Rad22-GFP as a marker for the presence of DNA damage repair foci (Noguchi *et al.* 2009). Although there were more Rad22-GFP foci in *new13*∆, there was a reduced number in *ptc1∆* and *SPAC607.02cΔ* cells compared with wild-type (Figure 4H), suggesting that these latter genes might play a role in assembly or maintenance of DNA repair foci during a normal S phase.

### Morphogenesis factors

**Figure 5:**
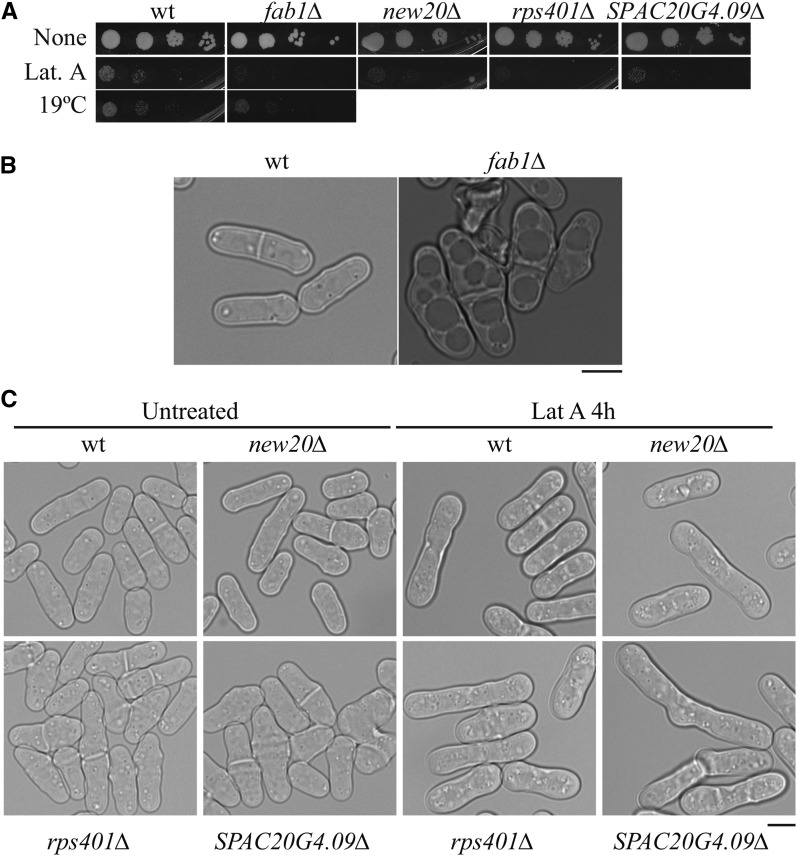
Characterization of gene deletion strains that are sensitive to actin cytoskeleton perturbation. (A) The 10-fold dilutions of cells from the indicated strains were spotted on YE agar in the absence or presence of 0.25 µM latrunculin A (Lat A). Plates were incubated at 29° or 19° for 3–8 d. (B) Wild-type (wt) and *fab1Δ* cells were grown at 32° in liquid YE to log phase. DIC images are shown. Scale bar, 5 µm. (C) DIC images of the indicated strains treated or untreated with Lat A for 4 hr at 32° are shown. Scale bar, 5 µm.

Ten gene deletions showed increased sensitivity to Lat A, which inhibits F-actin and blocks actomyosin ring formation and cytokinesis, or Lat A plus one additional factor (Figure 5A and Table S6). Fab1 is a 1-phosphatidylinositol-3-phosphate 5-kinase responsible for producing PI(3,5)P_2_ (McEwen *et al.* 1999). In *S. cerevisiae*, Fab1 is required for proper intracellular protein trafficking and vacuolar organization (Gary *et al.* 1998). Similarly, *S*. *pombe fab1∆* cells are short, stubby, and have large vacuoles (Figure 5B), as observed previously for point mutations of *fab1* (previously called *ste12* due to their sterility) (Morishita *et al.* 2002). Thus, it is likely that actin filament disruption exacerbated the decreased intracellular trafficking in *fab1Δ* cells, resulting in cell death.

Deletions of three other genes not previously linked to morphogenesis (*SPAC20G4.09∆*, *rps401∆*, and *new20∆*) also caused high sensitivity to Lat A (Figure 5A). *SPAC20G4.09∆* cells are obviously misshapen and become more so when treated with Lat A (Figure 5C). *rps401∆* cells vary slightly from normal morphology but do not show obvious morphological changes on Lat A treatment (Figure 5C). Despite severe Lat A sensitivity, *new20∆* cells appeared morphologically wild-type with or without Lat A treatment (Figure 5C). F-actin staining in these cells was consistent with their morphology in that F-actin patches were confined to areas of cell growth (Figure S3). More studies will be required to determine why these cells are so sensitive to actin disruption.

### New temperature-sensitive deletion strains

**Figure 6:**
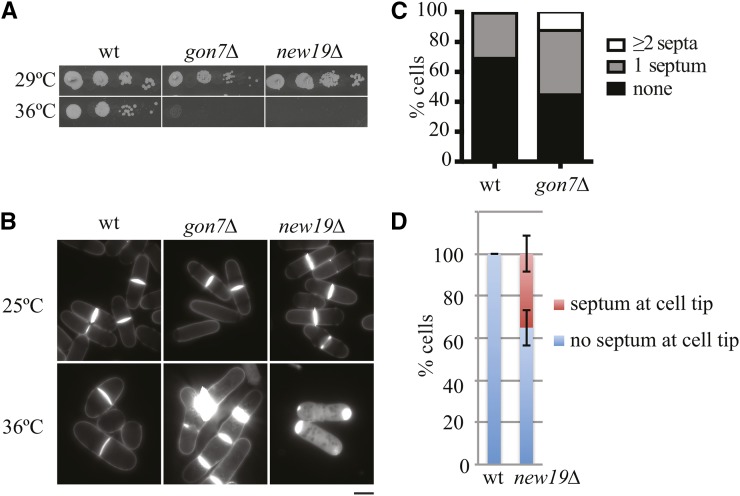
Characterization of temperature sensitive gene deletion strains. (A) The 10-fold dilutions of cells from the indicated strains were spotted on YE agar and plates were incubated at 29° or 36° for 2–3 d. (B) DAPI/calcofluor white staining images of wild-type (wt), *gon7Δ*, and *new19Δ* cells grown at 25° and 36° overnight. (C) Quantitation of the septum number of indicated strains grown at 36° overnight. (D) Quantitation of nonseptated cells with septum at the cell tips. Cells were grown at 36° overnight. Mean±SD from two separate experiments is shown. Scale bar, 5 µm.

The *gon7Δ* and *new19Δ* deletions were unable to survive when plated at 36° (Figure 6A and Table S7). Although *gon7Δ* cells had normal morphology at 25°, after growth at 36° overnight (16 hr) the cells became highly elongated and multi-septated (Figure 6, B and C). In *S. cerevisiae*, Gon7 is a component of the KEOPS complex that is required for t6A tRNA modification (Srinivasan *et al.* 2011) and promotes telomere uncapping and elongation (Downey *et al.* 2006), transcription (Kisseleva-Romanova *et al.* 2006), and chromosome segregation (Ben-Aroya *et al.* 2008). Although it is unclear how Gon7 impacts septation, loss of *GON7* in *S. cerevisiae* resulted in defects in N-linked oligosaccharide biosynthesis, and this may affect cell wall metabolism (Corbacho *et al.* 2004). The *new19Δ* cells are morphologically normal at 25°, but at 36° a large percentage divide without completely degrading their septum and septum remnants persist at new cell tips (Figures 6, B and D). New19 is the apparent ortholog of the *S. cerevisiae* signal peptidase complex component Spc1 (Wood *et al.* 2012); therefore, *new19* deletion is expected to disrupt protein targeting to the ER. The *new19* deletion may impair the targeting of primary septum degrading enzymes to the cell division site.

## Discussion

To expand the toolkits available for *S. pombe* genetic screens (Kim *et al.* 2010; Hayles *et al.* 2013; Gregan *et al.* 2006; Spirek *et al.* 2010; Rumpf *et al.* 2010), we used the *kanMX6* marker (Wach *et al.* 1994) to construct individual deletion strains of 281 genes. We assayed the growth of this cohort of strains under a battery of stresses, identifying new players in cell division, DNA damage response, and morphogenesis.

In some cases in which sensitivities of deletion strains have been reported, for example, with *chk1* and *hhp1* (Bimbo *et al.* 2005; Dhillon and Hoekstra 1994; Walworth *et al.* 1993), our data largely match the published literature, validating our methodology and providing confidence that the growth tests revealed new and useful information. Interestingly, there are some differences between sensitivities of various gene deletions between fission and budding yeast. For instance, deletion of the poly-ubiquitin gene, *ubi4*, did not significantly affect cell growth under any of the stress conditions tested; this is in contrast to loss of the orthologous gene in *S. cerevisiae*, which exhibits multiple phenotypes and stress sensitivities (Finley *et al.* 1987). One possible explanation for this difference is the presence of a fifth ubiquitin fusion gene in fission yeast, compared with only four in budding yeast, which could compensate for poly-ubiquitin loss.

During construction of the gene deletion strains, we unexpectedly found that a significant percentage of the G418-resistant colonies arising from transformation with the linear DNA fragment containing the *kanMX6* cassette contained a mixed population. In other words, PCR products indicative of both the correctly integrated *kanMX6* cassette and the ORF that had been targeted for deletion were detected. This perplexing result may be explained if the linear DNA fragment used for transformation was retained outside of the genome, with recombination and replacement of the ORF occurring only in a sub-population of the colony’s cells. We found that replicating the transformants to fresh YE-G418 plates at least three times and/or streaking to single colonies led to the elimination of cells containing the ORF in most cases. This observation may explain why certain deletion strains have been reported to retain the ORF that had been targeted for deletion, and yet be G418 resistant. Our careful analysis with three independent PCR reactions ensured the fidelity of this set of 281 strains.

Many of the genes targeted for deletion via this approach have been previously deleted with a similar approach (Hayles *et al.* 2013) or with other markers such as *ura4^+^*. For example, *nup124*, *btn1*, and *chk1* were deleted using other markers years ago (Chen *et al.* 2004; Gachet *et al.* 2005; Walworth *et al.* 1993). However, this deletion series contains many newly generated *kanMX6* deletions, expanding the list of nonessential *S. pombe* genes and genome-wide tools available for large-scale screens.

## Supplementary Material

Supporting Information

## References

[bib1] Alao J. P. Sunnerhagen P., 2008 Rad3 and Sty1 function in Schizosaccharomyces pombe: an integrated response to DNA damage and environmental stress? Mol. Microbiol. 68: 246–254. 18366437 10.1111/j.1365-2958.2008.06147.x

[bib2] Ayscough K. R. Stryker J. Pokala N. Sanders M. Crews P., 1997 High rates of actin filament turnover in budding yeast and roles for actin in establishment and maintenance of cell polarity revealed using the actin inhibitor latrunculin-A. J. Cell Biol. 137: 399–416. 9128251 10.1083/jcb.137.2.399 PMC2139767

[bib3] Azzalin, C.M., and J. Lingner, 2006 The human RNA surveillance factor UPF1 is required for S phase progression and genome stability. Current Biol. 16:433–439. 10.1016/j.cub.2006.01.018 16488880

[bib4] Bahler J. Wu J. Q. Longtine M. S. Shah N. G. McKenzie A. 3rd, 1998 Heterologous modules for efficient and versatile PCR-based gene targeting in *Schizosaccharomyces pombe*. Yeast 14: 943–951. 9717240 10.1002/(SICI)1097-0061(199807)14:10<943::AID-YEA292>3.0.CO;2-Y

[bib5] Baird D. Stefan C. Audhya A. Weys S. Emr S. D., 2008 Assembly of the PtdIns 4-kinase Stt4 complex at the plasma membrane requires Ypp1 and Efr3. J. Cell Biol. 183: 1061–1074. 19075114 10.1083/jcb.200804003 PMC2600738

[bib6] Beckouet F. Labarre-Mariotte S. Albert B. Imazawa Y. Werner M., 2008 Two RNA polymerase I subunits control the binding and release of Rrn3 during transcription. Mol. Cell. Biol. 28: 1596–1605. 18086878 10.1128/MCB.01464-07 PMC2258765

[bib7] Ben-Aroya S. Coombes C. Kwok T. O’Donnell K. A. Boeke J. D., 2008 Toward a comprehensive temperature-sensitive mutant repository of the essential genes of Saccharomyces cerevisiae. Mol. Cell 30: 248–258. 18439903 10.1016/j.molcel.2008.02.021 PMC4130347

[bib8] Beranek D. T., 1990 Distribution of methyl and ethyl adducts following alkylation with monofunctional alkylating agents. Mutat. Res. 231: 11–30. 2195323 10.1016/0027-5107(90)90173-2

[bib9] Bimbo A. Jia Y. Poh S. L. Karuturi R. K. den Elzen N., 2005 Systematic deletion analysis of fission yeast protein kinases. Eukaryot. Cell 4: 799–813. 15821139 10.1128/EC.4.4.799-813.2005 PMC1087820

[bib10] Bitton D. A. Wood V. Scutt P. J. Grallert A. Yates T., 2011 Augmented annotation of the Schizosaccharomyces pombe genome reveals additional genes required for growth and viability. Genetics 187: 1207–1217. 21270388 10.1534/genetics.110.123497 PMC3070528

[bib11] Carpy A. Krug K. Graf S. Koch A. Popic S., 2014 Absolute proteome and phosphoproteome dynamics during the cell cycle of Schizosaccharomyces pombe (Fission Yeast). *Molecular & cellular proteomics*. MCP 13: 1925–1936. 24763107 10.1074/mcp.M113.035824 PMC4125727

[bib12] Chang Y. F. Imam J. S. Wilkinson M. F., 2007 The nonsense-mediated decay RNA surveillance pathway. Annu. Rev. Biochem. 76: 51–74. 17352659 10.1146/annurev.biochem.76.050106.093909

[bib13] Chen X. Q. Du X. Liu J. Balasubramanian M. K. Balasundaram D., 2004 Identification of genes encoding putative nucleoporins and transport factors in the fission yeast Schizosaccharomyces pombe: a deletion analysis. Yeast 21: 495–509. 15116432 10.1002/yea.1115

[bib14] Chen, Z., S. McCrosky, W. Guo, H. Li, and J.L. Gerton, 2012 A genetic screen to discover pathways affecting cohesin function in Schizosaccharomyces pombe identifies chromatin effectors. G3 (Bethesda) 2:1161–1168. 10.1534/g3.112.003327 PMC3464108 23050226

[bib15] Corbacho I. Olivero I. Hernandez L. M., 2004 Identification of low-dye-binding (ldb) mutants of Saccharomyces cerevisiae. FEMS Yeast Res. 4: 437–444. 14734024 10.1016/S1567-1356(03)00162-4

[bib16] Costanzo M. Baryshnikova A. Myers C. L. Andrews B. Boone C., 2011 Charting the genetic interaction map of a cell. Curr. Opin. Biotechnol. 22: 66–74. 21111604 10.1016/j.copbio.2010.11.001

[bib17] Deshpande G. P. Hayles J. Hoe K. L. Kim D. U. Park H. O., 2009 Screening a genome-wide S. pombe deletion library identifies novel genes and pathways involved in genome stability maintenance. DNA Repair (Amst.) 8: 672–679. 19264558 10.1016/j.dnarep.2009.01.016 PMC2675035

[bib18] Dhillon N. Hoekstra M. F., 1994 Characterization of two protein kinases from Schizosaccharomyces pombe involved in the regulation of DNA repair. EMBO J. 13: 2777–2788. 8026462 10.1002/j.1460-2075.1994.tb06571.x PMC395157

[bib19] Dixon S. J. Costanzo M. Baryshnikova A. Andrews B. Boone C., 2009 Systematic mapping of genetic interaction networks. Annu. Rev. Genet. 43: 601–625. 19712041 10.1146/annurev.genet.39.073003.114751

[bib20] Downey M. Houlsworth R. Maringele L. Rollie A. Brehme M., 2006 A genome-wide screen identifies the evolutionarily conserved KEOPS complex as a telomere regulator. Cell 124: 1155–1168. 16564010 10.1016/j.cell.2005.12.044

[bib21] Finley D. Ozkaynak E. Varshavsky A., 1987 The yeast polyubiquitin gene is essential for resistance to high temperatures, starvation, and other stresses. Cell 48: 1035–1046. 3030556 10.1016/0092-8674(87)90711-2

[bib22] Gachet Y. Codlin S. Hyams J. S. Mole S. E., 2005 btn1, the Schizosaccharomyces pombe homologue of the human Batten disease gene CLN3, regulates vacuole homeostasis. J. Cell Sci. 118(Pt 23): 5525–5536. 16291725 10.1242/jcs.02656

[bib23] Gaits F. Shiozaki K. Russell P., 1997 Protein phosphatase 2C acts independently of stress-activated kinase cascade to regulate the stress response in fission yeast. J. Biol. Chem. 272: 17873–17879. 9211944 10.1074/jbc.272.28.17873

[bib24] Gary J. D. Wurmser A. E. Bonangelino C. J. Weisman L. S. Emr S. D., 1998 Fab1p is essential for PtdIns(3)P 5-kinase activity and the maintenance of vacuolar size and membrane homeostasis. J. Cell Biol. 143: 65–79. 9763421 10.1083/jcb.143.1.65 PMC2132800

[bib25] Gregan J. Rabitsch P. K. Rumpf C. Novatchkova M. Schleiffer A., 2006 High-throughput knockout screen in fission yeast. Nat. Protoc. 1: 2457–2464. 17406492 10.1038/nprot.2006.385 PMC2957175

[bib26] Hayles, J., V. Wood, L. Jeffery, K.L. Hoe, D.U. Kim *et al.*, 2013 A genome-wide resource of cell cycle and cell shape genes of fission yeast. Open Biol. 3: 130053. 10.1098/rsob.130053 PMC3866870 23697806

[bib27] Jourdain I. Brzezinska E. A. Toda T., 2013 Fission yeast Nod1 is a component of cortical nodes involved in cell size control and division site placement. PLoS ONE 8: e54142. 23349808 10.1371/journal.pone.0054142 PMC3547912

[bib28] Kelly F. D. Nurse P., 2011 Spatial control of Cdc42 activation determines cell width in fission yeast. Mol. Biol. Cell 22: 3801–3811. 21849474 10.1091/mbc.E11-01-0057 PMC3192860

[bib29] Kennedy, P.J., A.A. Vashisht, K.L. Hoe, D.U. Kim, H.O. Park *et al.*, 2008 A genome-wide screen of genes involved in cadmium tolerance in Schizosaccharomyces pombe. Toxicol. Sci. 106: 124–139. 10.1093/toxsci/kfn153 PMC2563147 18684775

[bib30] Kim D. U. Hayles J. Kim D. Wood V. Park H. O., 2010 Analysis of a genome-wide set of gene deletions in the fission yeast Schizosaccharomyces pombe. Nat. Biotechnol. 28: 617–623. 20473289 10.1038/nbt.1628 PMC3962850

[bib31] Kisseleva-Romanova E. Lopreiato R. Baudin-Baillieu A. Rousselle J. C. Ilan L., 2006 Yeast homolog of a cancer-testis antigen defines a new transcription complex. EMBO J. 25: 3576–3585. 16874308 10.1038/sj.emboj.7601235 PMC1538566

[bib32] Koressaar T. Remm M., 2007 Enhancements and modifications of primer design program Primer3. Bioinformatics 23: 1289–1291. 17379693 10.1093/bioinformatics/btm091

[bib33] Matia-Gonzalez A. M. Hasan A. Moe G. H. Mata J. Rodriguez-Gabriel M. A., 2013 Functional characterization of Upf1 targets in Schizosaccharomyces pombe. RNA Biol. 10: 1057–1065. 23619768 10.4161/rna.24569 PMC3904585

[bib34] Matsuyama A. Arai R. Yashiroda Y. Shirai A. Kamata A., 2006 ORFeome cloning and global analysis of protein localization in the fission yeast Schizosaccharomyces pombe. Nat. Biotechnol. 24: 841–847. 16823372 10.1038/nbt1222

[bib35] McEwen R. K. Dove S. K. Cooke F. T. Painter G. F. Holmes A. B., 1999 Complementation analysis in PtdInsP kinase-deficient yeast mutants demonstrates that Schizosaccharomyces pombe and murine Fab1p homologues are phosphatidylinositol 3-phosphate 5-kinases. J. Biol. Chem. 274: 33905–33912. 10567352 10.1074/jbc.274.48.33905

[bib36] Morishita, M., F. Morimoto, K. Kitamura, T. Koga, Y. Fukui *et al.*, 2002 Phosphatidylinositol 3-phosphate 5-kinase is required for the cellular response to nutritional starvation and mating pheromone signals in Schizosaccharomyces pombe. Genes Cells 7: 199–215. 10.1046/j.1356-9597.2001.00510.x 11895483

[bib37] Navarro F. J. Nurse P., 2012 A systematic screen reveals new elements acting at the G2/M cell cycle control. Genome Biol. 13: R36. 22624651 10.1186/gb-2012-13-5-r36 PMC3446289

[bib38] Noguchi E. Ansbach A. B. Noguchi C. Russell P., 2009 Assays used to study the DNA replication checkpoint in fission yeast. Methods Mol. Biol. 521: 493–507. 19563125 10.1007/978-1-60327-815-7_28 PMC3582217

[bib39] Pan X. Lei B. Zhou N. Feng B. Yao W., 2012 Identification of novel genes involved in DNA damage response by screening a genome-wide Schizosaccharomyces pombe deletion library. BMC Genomics 13: 662. 23173672 10.1186/1471-2164-13-662 PMC3536581

[bib40] Povirk L. F., 1996 DNA damage and mutagenesis by radiomimetic DNA-cleaving agents: bleomycin, neocarzinostatin and other enediynes. Mutat. Res. 355: 71–89. 8781578 10.1016/0027-5107(96)00023-1

[bib41] Rallis C. Lopez-Maury L. Georgescu T. Pancaldi V. Bahler J., 2014 Systematic screen for mutants resistant to TORC1 inhibition in fission yeast reveals genes involved in cellular ageing and growth. Biol. Open 3: 161–171. 24463365 10.1242/bio.20147245 PMC3925319

[bib42] Reid R. J. Gonzalez-Barrera S. Sunjevaric I. Alvaro D. Ciccone S., 2011 Selective ploidy ablation, a high-throughput plasmid transfer protocol, identifies new genes affecting topoisomerase I-induced DNA damage. Genome Res. 21: 477–486. 21173034 10.1101/gr.109033.110 PMC3044861

[bib43] Rumpf C. Cipak L. Novatchkova M. Li Z. Polakova S., 2010 High-throughput knockout screen in Schizosaccharomyces pombe identifies a novel gene required for efficient homolog disjunction during meiosis I. Cell Cycle 9: 1802–1808. 20404563 10.4161/cc.9.9.11526 PMC2883734

[bib44] Ryuko S. Ma Y. Ma N. Sakaue M. Kuno T., 2012 Genome-wide screen reveals novel mechanisms for regulating cobalt uptake and detoxification in fission yeast. *Molecular genetics and genomics*. MGG 287: 651–662. 22806344 10.1007/s00438-012-0705-9

[bib45] Slater M. L., 1973 Effect of reversible inhibition of deoxyribonucleic acid synthesis on the yeast cell cycle. J. Bacteriol. 113: 263–270. 4120066 10.1128/jb.113.1.263-270.1973 PMC251626

[bib46] Spirek M. Benko Z. Carnecka M. Rumpf C. Cipak L., 2010 S. pombe genome deletion project: an update. Cell Cycle 9: 2399–2402. 20519959 10.4161/cc.9.12.11914 PMC3132452

[bib47] Srinivasan M. Mehta P. Yu Y. Prugar E. Koonin E. V., 2011 The highly conserved KEOPS/EKC complex is essential for a universal tRNA modification, t6A. EMBO J. 30: 873–881. 21183954 10.1038/emboj.2010.343 PMC3049205

[bib48] Turi T. G. Webster P. Rose J. K., 1994 Brefeldin A sensitivity and resistance in Schizosaccharomyces pombe. Isolation of multiple genes conferring resistance. J. Biol. Chem. 269: 24229–24236. 7929079

[bib49] Ucisik-Akkaya, E., J.K. Leatherwood, and A.M. Neiman, 2014 A genome-wide screen for sporulation-defective mutants in Schizosaccharomyces pombe. G3 (Bethesda) 4: 1173–1182 10.1534/g3.114.011049 PMC4065261 24727291

[bib50] Untergasser A. Cutcutache I. Koressaar T. Ye J. Faircloth B. C., 2012 Primer3–new capabilities and interfaces. Nucleic Acids Res. 40: e115. 22730293 10.1093/nar/gks596 PMC3424584

[bib51] Wach A. Brachat A. Poehlmann R. Philippsen P., 1994 New heterologous modules for classical or PCR-based gene disruptions in Saccharomyces cerevisiae. Yeast 10: 1793–1808. 7747518 10.1002/yea.320101310

[bib52] Walworth N. Davey S. Beach D., 1993 Fission yeast chk1 protein kinase links the rad checkpoint pathway to cdc2. [see comments] Nature 363: 368–371. 8497322 10.1038/363368a0

[bib53] Wood V. Harris M. A. McDowall M. D. Rutherford K. Vaughan B. W., 2012 PomBase: a comprehensive online resource for fission yeast. Nucleic Acids Res. 40: D695–D699. 22039153 10.1093/nar/gkr853 PMC3245111

[bib54] Wu X. Chi R. J. Baskin J. M. Lucast L. Burd C. G., 2014 Structural insights into assembly and regulation of the plasma membrane phosphatidylinositol 4-kinase complex. Dev. Cell 28: 19–29. 24360784 10.1016/j.devcel.2013.11.012 PMC4349574

[bib55] Yamamoto M., 1980 Genetic analysis of resistant mutants to antimitotic benzimidazole compounds in Schizosaccharomyces pombe. *Molecular & general genetics*. MGG 180: 231–234. 7003311 10.1007/BF00267375

[bib56] Yarbro J. W., 1992 Mechanism of action of hydroxyurea. Semin. Oncol. 19(3, Suppl 9) 1–10. 1641648

[bib57] Zhang L. Ma N. Liu Q. Ma Y., 2013 Genome-wide screening for genes associated with valproic acid sensitivity in fission yeast. PLoS ONE 8: e68738. 23861937 10.1371/journal.pone.0068738 PMC3702616

[bib58] Zhou X. Ma Y. Fang Y., W. Gerile, W. Jaiseng *et al*, 2013 A genome-wide screening of potential target genes to enhance the antifungal activity of micafungin in Schizosaccharomyces pombe. PLoS ONE 8: e65904. 23738021 10.1371/journal.pone.0065904 PMC3667807

