# Supplementary material for: Identification of New Players in Cell Division, DNA Damage Response, and Morphogenesis Through Construction of Schizosaccharomyces pombe Deletion Strains
Source: G3 (Bethesda). 2014 Dec 31;5(3):361–70. doi: 10.1534/g3.114.015701 (PMC4349090; doi:10.1534/g3.114.015701)
Supplement: Supporting Information [file supp_5_3_361__index.html]

Identification of New Players in Cell Division, DNA Damage Response, and Morphogenesis Through Construction of Schizosaccharomyces pombe Deletion Strains — Supporting Information 

# Identification of New Players in Cell Division, DNA Damage Response, and Morphogenesis Through Construction of *Schizosaccharomyces pombe* Deletion Strains

## Supporting Information for Chen *et al.*, 2015

**Files in this Data Supplement:**

- Supporting Information - Figures S1-S3 and Tables S1-S7 (PDF, 325 KB)
- Figure S1 - Examples of growth assay sensitivities. (PDF, 188 KB)
- Figure S2 - Examples of PCR amplification products from the first round of PCR reactions. (PDF, 164 KB)
- Figure S3 - F-actin staining of LatA sensitive strains. (PDF, 148 KB)
- Table S1 - .xlsx, 44 KB
- Table S2 - Sequences of common oligos. (.xlsx, 8 KB)
- Table S3 - Summary of sensitivities of the deletions to different stress conditions. (.xlsx, 16 KB)
- Table S4 - Sensitive to ≥3 conditions. (.xlsx, 12 KB)
- Table S5 - Deletions which are sensitive to drugs interfering with DNA metabolism. (.xlsx, 11 KB)
- Table S6 - Deletions which are sensitive to actin disrupting agent latrunculin A. (.xlsx, 9 KB)
- Table S7 - Sensitive to high temperature only or plus one additional sensitivity. (.xlsx, 8 KB)
