# Supplementary material for: Identification of New Players in Cell Division, DNA Damage Response, and Morphogenesis Through Construction of Schizosaccharomyces pombe Deletion Strains
Source: G3 (Bethesda). 2014 Dec 31;5(3):361–70. doi: 10.1534/g3.114.015701 (PMC4349090; doi:10.1534/g3.114.015701)
Supplement: Supporting Information [file supp_g3.114.015701_015701SI.pdf]

G3· Genes | Genomes | Genetics

Identification of new players in cell division, DNA damage response, and morphogenesis through construction of *Schizosaccharomyces pombe* deletion strains

Jun-Song Chen[*], Janel R. Beckley[*], Nathan A. McDonald[*], Liping Ren[*], MariaSanta Mangione[*], Sylvia J. Jang[*], Zachary C. Elmore[*], Nicole Rachfall[*], Anna Feoktistova[*], Christine M. Jones, Alaina H. Willet[*], Rodrigo Guillen[*], Danny A. Bitton[§], Jürg Bähler[§], Michael A. Jensen[¶], Nick Rhind[‡], and Kathleen L. Gould[*]

[*]Department of Cell and Developmental Biology, Vanderbilt University School of Medicine, Nashville, TN 37232, USA; [§]Department of Genetics, Evolution and Environment and UCL Cancer Institute, University College London, London WC1E 6BT, UK; [¶]Genome technology Center, Stanford University School of Medicine, Palo Alto, CA 01605, USA; [‡]Department of Biochemistry and Molecular Pharmacology, University of Massachusetts Medical School, Worcester, MA 01605, USA

Corresponding author:
Kathleen L. Gould
Department of Cell and Developmental Biology
Vanderbilt University School of Medicine
Nashville, TN 37232
USA
615-343-9502
kathy.gould@vanderbilt.edu

DOI: 10.1534/g3.114.015701

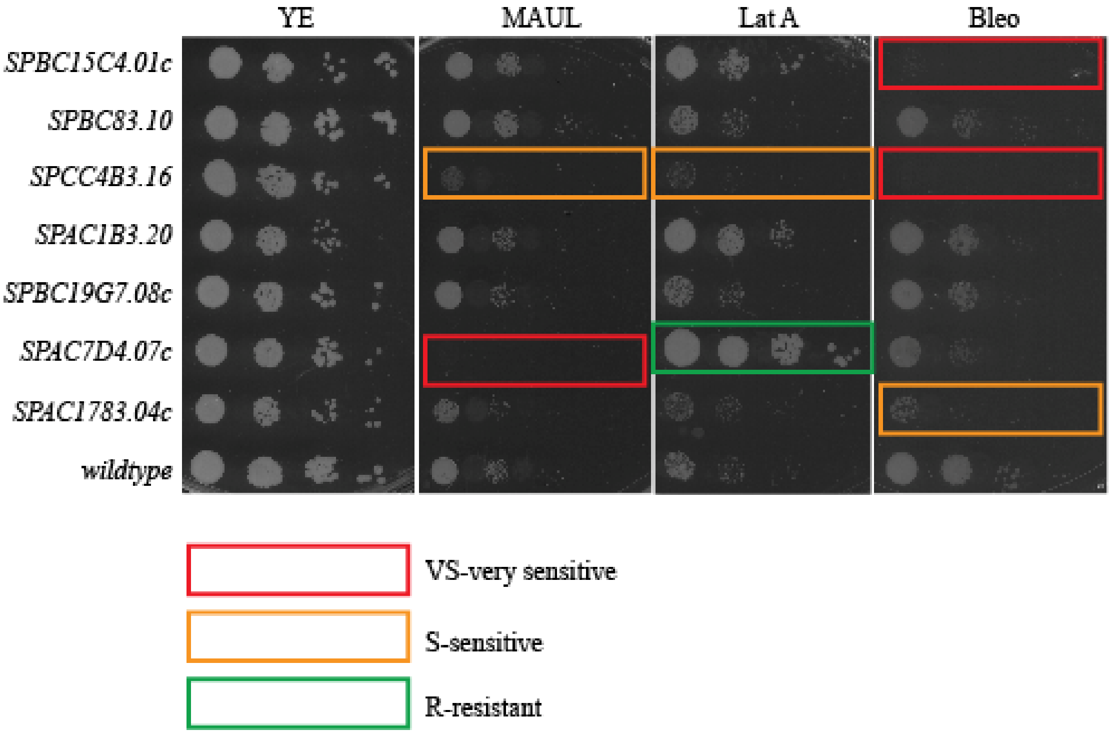

Figure S1 Examples of growth assay sensitivities. An example set of strains tested for growth at 29˚C on YE, minimal medium (MAUL), YE+LatA, and YE+bleomycin (Bleo) with 10-fold serial dilutions. Representative scoring of growth as very sensitive (VS), sensitive (S), and resistant (R) is indicated with colored boxes.

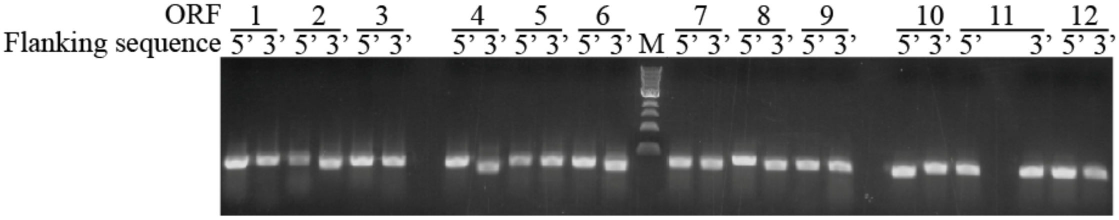

Figure S2 Examples of PCR amplification products from the first round of PCR reactions. 5 μL of each first round PCR reaction was loaded on 0.8% agarose gel for visualization. M, 1 kb DNA ladder (the bottom band is 500 bp).

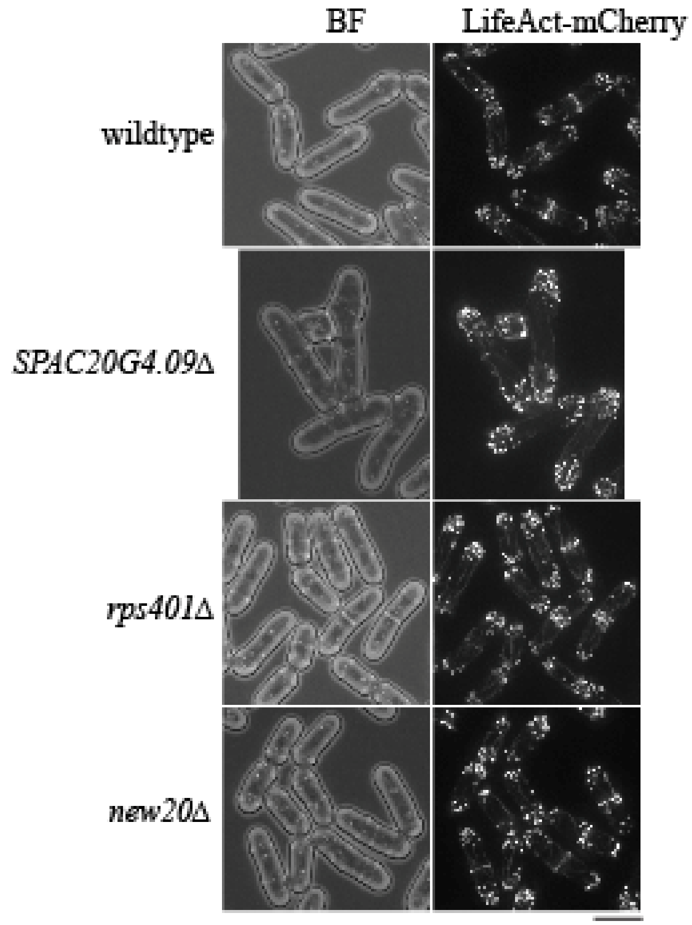

Figure S3 F-actin staining of LatA sensitive strains. The distribution of F-actin was detected in the indicated strains using an integrated version of LifeAct-mCherry. Representative live cell images are shown. BF, bright field; Scale bar, 5 µm.

Tables S1-S7
Available for download as Excel files at http://www.g3journal.org/lookup/suppl/doi:10.1534/g3.114.015701/-/DC1

Table S1

Table S2 Sequences of common oligos

Table S3 Summary of sensitivities of the deletions to different stress conditions

Table S4 Sensitive to ≥3 conditions

Table S5 Deletions which are sensitive to drugs interfering with DNA metabolism

Table S6 Deletions which are sensitive to actin disrupting agent latrunculin A

Table S7 Sensitive to high temperature only or plus one additional sensitivity
