# Supplementary material for: Identification of New Players in Cell Division, DNA Damage Response, and Morphogenesis Through Construction of Schizosaccharomyces pombe Deletion Strains
Source: G3 (Bethesda). 2014 Dec 31;5(3):361–70. doi: 10.1534/g3.114.015701 (PMC4349090; doi:10.1534/g3.114.015701)
Supplement: Supporting Information [file supp_g3.114.015701_FigureS1.pdf]

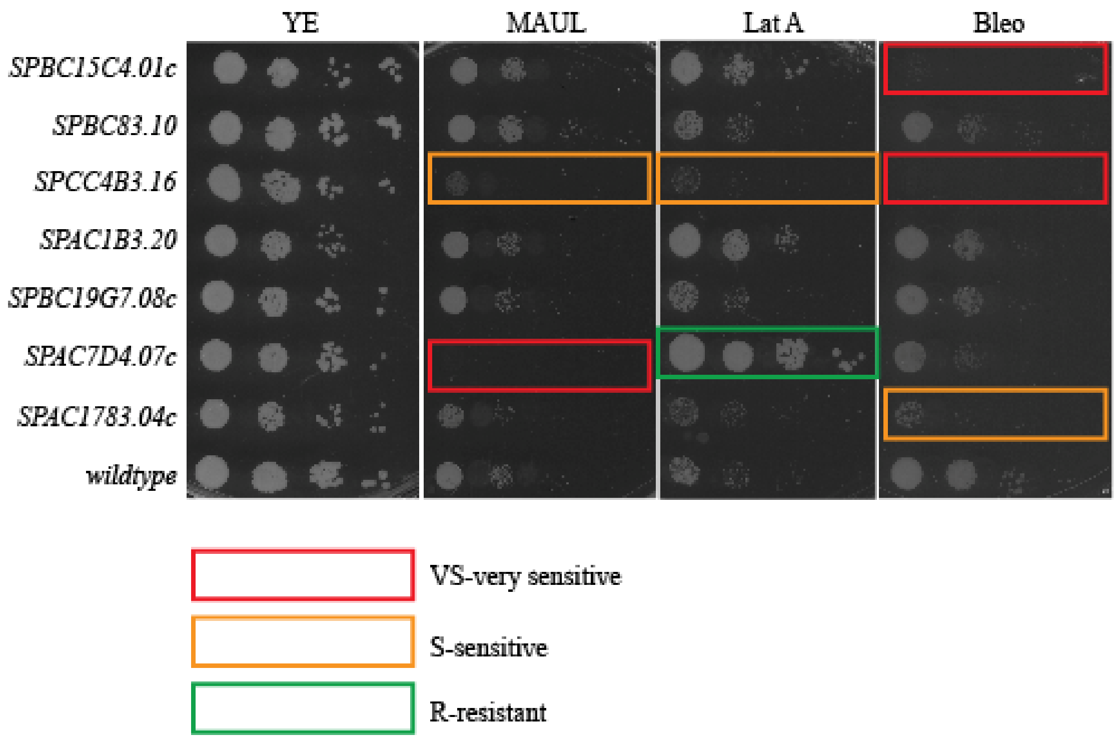

Figure S1 Examples of growth assay sensitivities. An example set of strains tested for growth at 29˚C on YE, minimal medium (MAUL), YE+LatA, and YE+bleomycin (Bleo) with 10-fold serial dilutions. Representative scoring of growth as very sensitive (VS), sensitive (S), and resistant (R) is indicated with colored boxes.
