# Supplementary material for: Identification of New Players in Cell Division, DNA Damage Response, and Morphogenesis Through Construction of Schizosaccharomyces pombe Deletion Strains
Source: G3 (Bethesda). 2014 Dec 31;5(3):361–70. doi: 10.1534/g3.114.015701 (PMC4349090; doi:10.1534/g3.114.015701)
Supplement: Supporting Information [file supp_g3.114.015701_FigureS2.pdf]

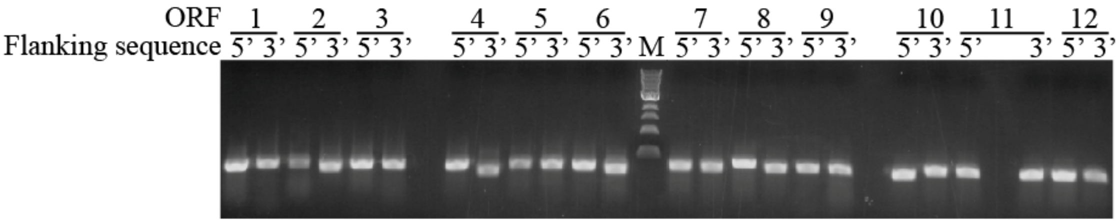

Figure S2 Examples of PCR amplification products from the first round of PCR reactions. 5 µL of each first round PCR reaction was loaded on 0.8% agarose gel for visualization. M, 1 kb DNA ladder (the bottom band is 500 bp).
