# Supplementary material for: Identification of New Players in Cell Division, DNA Damage Response, and Morphogenesis Through Construction of Schizosaccharomyces pombe Deletion Strains
Source: G3 (Bethesda). 2014 Dec 31;5(3):361–70. doi: 10.1534/g3.114.015701 (PMC4349090; doi:10.1534/g3.114.015701)
Supplement: Supporting Information [file supp_g3.114.015701_FigureS3.pdf]

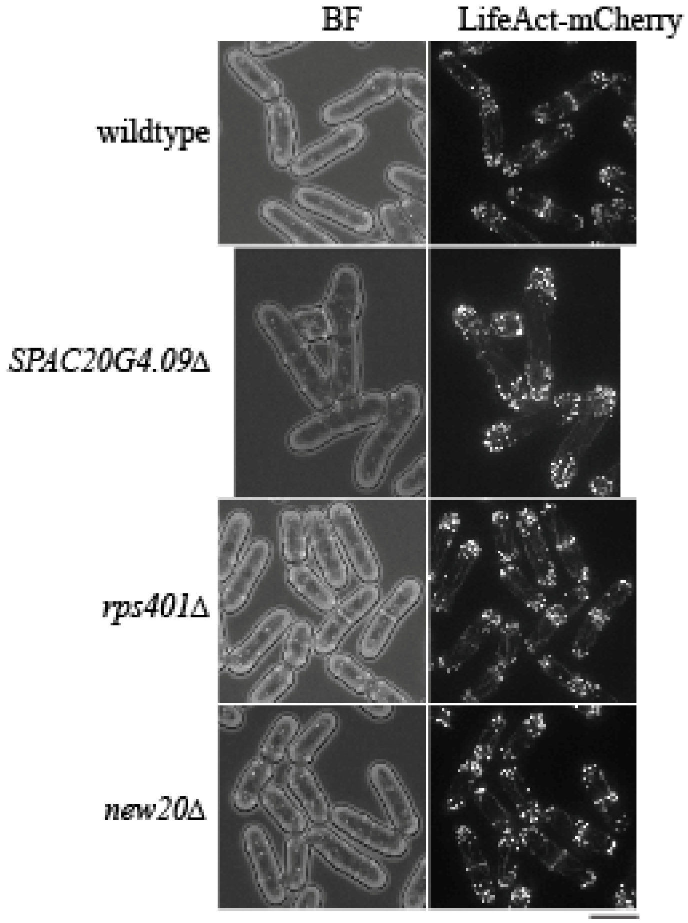

Figure S3 F-actin staining of LatA sensitive strains. The distribution of F-actin was detected in the indicated strains using an integrated version of LifeAct-mCherry. Representative live cell images are shown. BF, bright field; Scale bar, 5 µm.
